# What role does compassion have on quality care ratings? A regression analysis and validation of the SCQ in emergency department patients

**DOI:** 10.1186/s12873-024-01040-8

**Published:** 2024-07-18

**Authors:** Harrison Boss, Cara MacInnis, Roland Simon, Jeanette Jackson, Markus Lahtinen, Shane Sinclair

**Affiliations:** 1 Department of Psychology, University of Calgary, Calgary, Canada; 2 Department of Psychology, Acadia University, Wolfville, Canada; 3 Health Quality Council of Alberta, Calgary, Canada; 4 Faculty of Nursing, University of Calgary, Calgary, Canada; 5 Compassion Research Lab, University of Calgary, Calgary, Canada

**Keywords:** Compassion, Compassionate Care, Quality care, Emergency Medicine, Patient Experience, Measurement

## Abstract

**Objective:**

To examine the unique contribution of patient reported experiences of compassion to overall patient quality care ratings. Additionally, we assess whether patients’ reported experiences of compassion in the emergency department differed between sociodemographic groups.

**Methods:**

Provincial data for this cross-sectional study were collected from 03/01/2022 to 09/05/2022 from 14 emergency departments in Alberta, Canada. Data from 4501 emergency department patients (53.6% women, 77.1% White/European) were analyzed. The primary outcome was patients’ overall quality care ratings during their most recent ED visit. Measures included in the hierarchical stepwise regression included demographics, and those drawn from the Emergency Department Patient Experience of Care (EDPEC) questionnaire: single and multi-item measures of patient information (e.g., patient perceptions health) and patient experience (e.g., physician communication), and compassion (e.g., Sinclair Compassion Questionnaire; SCQ-ED).

**Results:**

Data from 4501 ED patients were analysed. Stepwise hierarchical linear multiple regression indicated that of 21 included variables, compassion most strongly predicted overall quality care ratings (b=1.61, 95% CI 1.53-1.69, *p*<.001, *f*^*2*^=.23), explaining 19% unique variance beyond all other measures. One-way ANOVAs indicated significant demographic differences in mean compassion scores, such that women (*vs.* men) reported lower compassion (MD=-.15, 95% CI=-.21, -.09, *p*<.001), and Indigenous (*vs.* White) patients reported lower compassion (MD=-.17, 95% CI =-.34, -.01, *p*=.03).

**Conclusions:**

Compassion was identified as a key contributor to ED overall quality care ratings, and experiences of compassion varied as a function of demographics. Patient-reported compassion is an indicator of quality care that needs to be formally integrated into clinical care and quality care assessments.

**Supplementary Information:**

The online version contains supplementary material available at 10.1186/s12873-024-01040-8.

## Introduction

In comparison to other healthcare environments, emergency care is relatively unmatched when it comes to utilization, pace, and acuity. In light of these factors, and the increasing demand of patients seeking care in emergency departments (EDs), there have been urgent calls [1–4] to ensure that patients receive care that is both safe and high-quality. Increasingly, a recognized critical component of quality care is compassion [5–7]. In this study, we examine how compassion contributes to a primary outcome of the patient experience, overall quality care rating, and whether there are demographic differences in patients’ experiences of compassion. We measure compassion using a validated patient reported measure adapted for use in the ED.

Compassion is operationalized as “a virtuous response that seeks to address the suffering and needs of a person through relational understanding and action.”[5 p.195] Compassion has potential to alleviate patient suffering and contribute to improved quality care [5, 7–9]. Likewise, less compassionate care may promote negative perceptions of care, patient complaints, and worse patient outcomes [1–5, 7–11]. Unfortunately, patients in various healthcare settings have indicated that compassion is one of their greatest unmet needs, and there are indications that this is increasing [2, 3, 7, 9, 10]. The implications of health systems that are lacking compassion on patients and healthcare provider well-being are well-documented [2, 3, 11]. In the ED specifically, a lack of compassion was identified as a primary factor in system wide increased patient safety issues, adverse medical events, and in some cases mortality [4].

A mixed-methodological study examining the role of compassion in emergency medicine settings found that UK National Health Service consultants who reported higher levels of compassion fatigue and/or lower levels of compassion satisfaction reported a greater propensity to treat both colleagues and patients with irritability, and with a reduced standard of care [1]. Although these findings are important steps to understanding the consequences of deficiencies in compassion, more research examining patient experiences of compassion in the ED is needed.

Recently, a valid and reliable patient reported compassion measure was developed, providing the opportunity to investigate the impact of compassion in the ED setting. The Sinclair Compassion Questionnaire’s (SCQ) validity has been established in long term care, hospice, and acute care settings [12] and was recently identified as the standard for evaluating compassion in research and clinical care [6]. In the current study, we verify its validity in the ED, and explore two principal goals. First, we examine whether the SCQ-ED explains variance in overall quality care ratings. Specifically, whether compassion predicts unique variance in overall quality care ratings, over and above other commonly collected measures of patient information, demographics, and patient experience. Second, we explore whether ED patients’ experiences of compassion varied based on demographics, with the aim of determining whether any specific groups of patients indicated differences in the compassion they received in the ED.

## Methods

### Study design, procedure, and sample

Employing a cross-sectional survey design, data were collected from ED patients between 03/01/2022 to 09/05/2022 in Alberta, Canada. Data were collected from the fourteen busiest urban and regional hospital EDs. Every two weeks, 160 patients were sampled from each ED (50% of which were discharged from the ED, and 50% who were admitted to the hospital after their ED visit). The survey vendor called respondents up to 9 times, at various times and days in an attempt to reach them. Surveying continued until at least 20 discharged and 20 admitted patients from each of the 14 EDs have responded, on each bi-weekly wave. Data from a total of 4501 ED patients were available for analysis.

Exclusion criteria were patients who: were under 16; left the ED without being seen or treated; died in the context of their ED visit or inpatient admission; were without contact information (i.e., phone number), had privacy-sensitive cases (e.g., domestic abuse, attempted suicide, etc.), or had been surveyed in the past year. The patient response rate during the study period was 23%.

This study is reported in accordance with the Strengthening the Reporting of Observational Studies in Epidemiology (STROBE) reporting guidelines for cross-sectional studies. This study was approved the Research Ethics Board (CHREB) at the University of Calgary (REB21-1938). In accordance with our REB approval, we were granted a waiver of consent for all participants, as gaining the contact information for these individuals through this administrative data was deemed not feasible. The dataset was obtained from the data custodians, the Health Quality Council of Alberta.

### Measures

#### Demographic and patient information

Demographics including age, gender, education, ethnicity, household income, self-reported overall health, self-perceived financial situation, and reason for visit were included (See Appendix A for full demographic questions).

#### Emergency department patient experience of care

Seventeen items from the Emergency Department Patient Experience of Care (EDPEC) survey, which has been extensively used and validated [13–15], was used to assess patients’ experiences during their most recent ED visit; referred hereafter as patient experience measures. Domains of patient experience included (See Appendix A): multi-item composites such as communication with patients by ED physicians (current sample Cronbach’s α = .83; Q17-20), communication with patients by ED nurses (current sample Cronbach’s α = .82; Q13-16), and single item measures covering: whether the patient arrived by ambulance (Q3), timeliness of care (Q4), new medication received (Q5), whether the side effects of new medications were described in a clear way (Q6), whether patient had pain (Q7), whether doctors and nurses attempted to reduce patient pain (Q8), pain medication received (Q9), whether the possible side effects of pain meds were clearly described (Q10), whether common tests were conducted (Q11), whether doctors and nurses provided sufficient info on the results of tests (Q12).

Finally, our primary outcome measure queried patients’ overall perceptions of care (i.e., 0 corresponding to the worst care possible, and 10 to the best care) during their most recent ED visit (Q21), referred to hereafter as overall quality care ratings.

#### Sinclair compassion questionnaire

The SCQ [12] measures a single latent construct of compassion and demonstrates strong internal consistency (original study Cronbach’s ***α*** = .96). The development of the SCQ rigorously adhered to measure development guidelines [16, 17] and heavily featured patient perspectives throughout its development. The full 15-item SCQ (current sample Cronbach’s ***α*** = .98) was included in this study, querying patients’ experiences of compassion by their healthcare providers (HCP) during their most recent ED visit on a 5-point Likert scale. The SCQ-ED, along with a variety of adapted versions (e.g., SCQ-Short Form, SCQ-Primary Care, etc.) are freely available to researchers and clinicians at www.compassionmeasure.com.

#### EQ-5D

The EQ-5D is a 5-item measure extensively used and validated non-disease specific tool aimed at measuring patients’ quality of life (current sample Cronbach’s ***α*** = .77) [18]. Full wording of the EQ-5D can be found in Appendix A.

### Data analysis

After preparing the dataset for analyses (e.g., data cleaning), the following analyses were conducted to address the study objectives. First, validity of the SCQ-ED in this population was verified (see Appendix B). Next, we assessed correlations (Pearson and point-biserial) between compassion and demographics, patient information, and patience experience variables. This served as a preliminary exploration of the zero-order relationships before moving on to the regression analysis addressing our primary objective. To explore whether and how much compassion uniquely predicted the variance explained for patients’ overall quality care ratings beyond patient demographics and information as well as patience experience measures contained in the EDPEC, we conducted a stepwise hierarchical linear multiple regression. It is important to note that throughout this study we will use terminology such as “predict” or “predictive”, as it normatively used in regression-based analyses. However, such language should not be conflated with etiological or causal claims, rather it refers to multiple factors that in together predict an outcome of interest, regardless of causality [19]. This regression analysis provided an opportunity to directly compare the explanatory power of all variables for quality care ratings simultaneously through the standardization of the beta weights. Finally, we sought to address whether any specific groups of patients reported lower levels of compassion during their recent ED visits by conducting analysis of variance (ANOVAs) with patient demographics.

#### Pearson and point-biserial correlations

Pearson or point-biserial correlation analyses were conducted between all continuous and dichotomous variables. These analyses provide information about the extent to which two variables are related to each other linearly, ranging between -1 and 1.A common rule of thumb for interpreting the strength of correlations states that coefficients of .10 indicate small associations, .30 indicate moderate associations, and those .50 or greater indicate large associations [20].

#### Multiple linear regression

A three-step hierarchical linear regression was conducted with overall quality care ratings as the outcome measure. Variables were added in a stepwise manner to assess the unique contribution of demographics and patient information (i.e., EQ-5D) [Step 1], patient experience measures (i.e., EDPEC) [Step 2], and compassion (i.e., SCQ-ED) [Step 3] on overall quality care ratings. This strategy also allowed for the direct comparison of the contribution of each of these variables through the standardization of the beta-weights (i.e., B in Table 4).

We utilized Cohen’s *f*^2^ as an indicator of local effect size for our multiple regressions. The common rule of thumb of interpreting *f*^2^ indicates that .02 = small, .15 = medium, and .35 = a large effect [20].

#### Analysis of variance

Analysis of variance (ANOVA) was used to explore whether SCQ-ED scores differed based on categorical demographic variables. We have used η^2^ as an indicator of effect size (.01 = small, .06 = medium, and .14 = large effects [21]. Post-hoc analyses using Tukey’s HSD [22] were used to assess pairwise comparisons.

## Results

A total of 4501 patients completed the survey, gender characteristics consisted of 53.6% women (45.3% men). Ethnicity of the sample was predominantly White/European (77.1%). The sample age was well distributed across all groups, with 16–24-year-olds representing the smallest proportion of the sample (9.3%), and 65-74-year-olds representing the largest proportion (16.2%). Full sample characteristics can be found in Table 1. All analyses were conducted using IBM SPSS 27.

**Table 1:** Sample characteristics

**Gender**	
Men	2039 (45.3%)
Women	2413 (53.6%)
Non-Binary	13 (.3%)
Transgender	9 (.2%)
Self Described Other Gender	2 (<.1%)
**Total (N)**	4476
**Age (Years of Age)**	
16-24	418 (9.3%)
25-34	672 (14.9%)
35-44	695 (15.4%)
45-54	584 (13.0%)
55-64	724 (16.1%)
65-74	729 (16.2%)
75+	617 (13.7%)
**Total (N)**	4468
**Ethnicity**	
White/European	3133 (77.1%)
Indigenous Peoples of Canada	221 (5.4%)
South Asian	221 (5.4%)
Southeast Asian	174 (3.9%)
East Asian	100 (2.5%)
Latin American/South American/Hispanic	98 (2.4%)
Other ethnicities	36 (.9%)
Mixed	79 (1.9%)
**Total (N)**	4062

Brackets indicate percentage of total sample. Although included as response options, no participants identified as Black or African American, or Middle Eastern, North African, and West African

### Preliminary analyses

Listwise deletion was employed for missing data (*n* = 304, see Appendix B for missing value analysis). The SCQ-ED demonstrated excellent validity [6, 12] and internal consistency/reliability, with a Cronbach’s alpha of .98 (*N* = 4197). Descriptive statistics for the SCQ-ED can be found in Table 2, along with SCQ-ED item intercorrelations in Appendix C.

**Table 2:** Descriptive Statistics for the SCQ Emergency Department

Item	N	Min	Max	Mean	Std. Dev.	Skewness	Kurtosis
Made me feel cared for	4462	1	5	4.24	.91	-1.59	2.73
Genuine concern	4466	1	5	4.22	.91	-1.49	2.32
Communicated sensitively	4465	1	5	4.30	.85	-1.66	3.38
Attentive	4459	1	5	4.20	.93	-1.44	2.00
Provided me with comfort	4451	1	5	4.15	.97	-1.35	1.54
Very supportive	4460	1	5	4.25	.90	-1.51	2.46
Provided care in gentle manner	4458	1	5	4.33	.81	-1.67	3.69
Spoke with kindness	4468	1	5	4.36	.79	-1.69	3.89
Saw as person	4416	1	5	4.18	.97	-1.40	1.71
Behaved in caring way	4468	1	5	4.30	.85	-1.60	3.05
Really understood needs	4446	1	5	4.15	.98	-1.35	1.53
Good relationship	4432	1	5	4.20	.92	-1.35	1.80
See from my perspective	4363	1	5	4.08	1.01	-1.26	1.19
Warm presence	4431	1	5	4.17	.91	-1.36	1.86
Sincere	4434	1	5	4.27	.86	-1.53	2.76

*N* = 4416-4468

### Exploring the zero-order relationships

Pearson and point-biserial correlational analyses revealed a large number of statistically significant relationships. Mean SCQ-ED scores were strongly associated with overall quality care ratings (*r* = .76, *p* <.001), nursing communication (*r* = .61, *p* = <.001), and doctor communication (*r* = .56, *p* <.001). Moderate relationships were revealed between the SCQ-ED and whether doctors and nurses provided sufficient information about the results of tests conducted (*r* = .38, *p* <.001), and whether doctors and nurses attempted to reduce patients’ pain (*r* = .33, *p* <.001). Small to moderate relationships also existed between the SCQ-ED and whether patients received care within 30 minutes (*r* = .26, *p* <.001), whether doctors or nurses clearly described the potential side effects of new medications (*r* = .26, *p* <.001), and whether the potential side effects of pain medications were discussed (*r* = .22, *p* <.001). Additional correlational relationships between the SCQ-ED, EDPEC items, and the EQ5D can be found in Table 3, and full correlational relationships, including demographics can be found in Appendix D.

**Table 3:** Summary of Pearson and Point-Biserial Correlations

		SCQ-ED	Care Rating	Nurse Comm	Doctor Comm	Care in 30 Mins	EQ-5D Mean	Health Today	Overall Health	Had Pain	Tried to Reduce Pain	Pain Meds	Pain Med Side Eff Discussed	Had Tests	Test Info Discussed	New Meds	New Meds Discussed
SCQ-ED	*r*																
	*N*	4485															
Care Rating	*r*	.76^***^	--														
	*N*	4455	4468														
Nurse Comm	*r*	.61^***^	.57^***^	--													
	*N*	4432	4412	4442													
Doctor Comm	*r*	.56^***^	.51^***^	.54^***^	--												
	*N*	4367	4349	4341	4376												
Care in 30 Mins	*r*	.26^***^	.32^***^	.21^***^	.15^***^	--											
	*N*	4349	4330	4308	4246	4362											
EQ-5D Mean	*r*	-.13^***^	-.12^***^	-.15^***^	-.15^***^	.04^*^	--										
	*N*	4452	4441	4410	4346	4329	4467										
Health Today	*r*	.15^***^	.16^***^	.15^***^	.14^***^	-.02^***^	-.60^***^	--									
	*N*	4259	4255	4224	4162	4142	4270	4273									
Overall Health	*r*	.18^***^	.16^***^	.16^***^	.16^***^	-.02	-.47^***^	.59^***^	--								
	*N*	4418	4410	4376	4313	4298	4412	4243	4433								
Had Pain	*r*	-.08^***^	-.10^***^	-.07^***^	-.07^***^	-.10^***^	.09^***^	-.01	.01	--							
	*N*	4447	4430	4404	4338	4333	4428	4239	4395	4462							
Tried to Reduce Pain	*r*	.33^***^	.35^***^	.27^***^	.22^***^	.24^***^	-.03	.01	.04^*^	.^c^	--						
	*N*	3056	3042	3027	3007	2981	3039	2902	3027	3050	3067						
Pain Meds	*r*	.17^***^	.18^***^	.15^***^	.11^***^	.11^***^	.04^*^	-.02	-.02	.^c^	.55^***^	--					
	*N*	3029	3014	3000	2980	2953	3012	2875	2998	3019	3005	3039					
Pain Med Side Eff Discussed	*r*	.22^***^	.21^***^	.23^***^	.18^***^	.07^**^	-.10^***^	.09^***^	.11^***^	.^c^	.18^***^	.^c^	--				
	*N*	1677	1668	1671	1652	1639	1671	1590	1654	1676	1672	1661	1683				
Had Tests	*r*	.10^***^	.11^***^	.08^***^	.05^***^	.07^***^	.05^**^	-.02	-.03	.11^***^	.11^***^	.13^***^	.02	--			
	*N*	4424	4406	4382	4318	4307	4405	4215	4371	4407	3032	3006	1662	4439			
Test Info Discussed	*r*	.38^***^	.37^***^	.33^***^	.41^***^	.15^***^	-.09^***^	.07^***^	.10^***^	-.04^*^	.22^***^	.10^***^	.20^***^	.^c^	--		
	*N*	3568	3555	3541	3505	3468	3556	3407	3526	3555	2533	2510	1458	3561	3580		
New Meds	*r*	.07^***^	.08^***^	.05^**^	.03^*^	.08	.01	-.01	.03	.06^***^	.26^***^	.30^***^	.03	.09^***^	.00	--	
	*N*	4300	4282	4259	4195	4197	4282	4095	4246	4282	2944	2927	1601	4263	3418	4313	
New Meds Discussed	*r*	.26^***^	.21^***^	.24^***^	.22^***^	.08^**^	-.09^**^	.05	.06	-.04	.18^***^	.03	.69^***^	.04	.26^***^	.^c^	--
	*N*	1143	1145	1143	1131	1127	1145	1104	1134	1142	834	837	647	1139	983	1149	1149

*N* = 4485-647. Pearson and point biserial correlations are denoted with *r.* ^***^p <.001, ^**^p <.01, ^*^p <.05

^c^denotes values that cannot be computed because at least one of the variables is constant

### Assessing the unique contribution of compassion to quality care ratings

A three-step hierarchical multiple regression was conducted to predict overall quality care ratings from applicable continuous, ordinal, and dichotomously coded variables (*N* = 2998 Table 4).

**Table 4:** Stepwise multiple regression results for overall quality care ratings outcome

Step 1 (Demographics)	b	SE	B	*t*	*p*	95% CI	*f*^2^
Constant	6.17	.39		15.96	<.001	[5.41, 6.93]	
Age	.20	.02	.18	9.38	<.001	[.16, .24]	.03
Ethnicity	.06	.10	.01	.64	.521	[-.13, .26]	.00
Gender	-.21	.07	-.05	-2.84	.005	[-.35, -.06]	.00
Education	-.04	.02	-.04	-2.08	.038	[-.08, .00]	.00
Born in Canada	.11	.10	.02	1.07	.283	[-.09, .31]	.00
Financial Situation	.01	.03	.01	.42	.678	[-.05, .08]	.00
Health Today	.02	.00	.15	6.17	<.001	[.01, .02]	.01
Current Pain	-.37	.08	-.08	-4.63	<.001	[-.52, -.21]	.01
EQ5D Pain	-.13	.05	-.06	-2.82	.005	[-.23, -.04]	.00
EQ5D Anxiety/Depression	-.05	.04	-.02	-1.18	.238	[-.14, .03]	.00
EQ5D Self Care	-.01	.07	.00	-.13	.899	[-.15, .13]	.00
EQ5D Mobility	.07	.05	.03	1.31	.189	[-.03, .18]	.00
EQ5D Usual Activities	.03	.05	.01	.57	.568	[-.07, .12]	.00
Overall health	.15	.04	.08	3.48	<.001	[.06, .23]	.00
Step 2 (Experience)	b	SE	B	*t*	*p*	95% CI	*f*^2^
Constant	-2.23	.37		-6.07	<.001	[-2.96, -1.51]	
Age	.13	.02	.12	7.51	<.001	[.10, .16]	.01
Ethnicity	.13	.08	.03	1.74	.082	[-.02, .28]	.00
Gender	.02	.06	.01	.37	.712	[-.09, .13]	.00
Education	-.04	.02	-.04	-2.50	.012	[-.07, -.01]	.00
Born in Canada	-.01	.08	.00	-.18	.854	[-.17, .14]	.00
Financial Situation	-.01	.02	-.01	-.49	.621	[-.06, .04]	.00
Health Today	.01	.00	.10	5.21	<.001	[.01, .02]	.00
Current Pain	-.22	.06	-.05	-3.53	<.001	[-.34, -.10]	.00
EQ5D Pain	-.08	.04	-.04	-2.11	.035	[-.15, -.01]	.00
EQ5D Anxiety/Depression	.03	.03	.01	.85	.397	[-.04, .09]	.00
EQ5D Self Care	.05	.05	.01	.83	.405	[-.06, .15]	.00
EQ5D Mobility	.05	.04	.02	1.21	.228	[-.03, .13]	.00
EQ5D Usual Activities	.01	.04	.01	.30	.761	[-.06, .08]	.00
Overall health	.07	.03	.04	2.23	.026	[.01, .14]	.00
**Nurse Communication**	**1.17**	**.05**	**.37**	**22.47**	**<.001**	**[1.07, 1.27]**	**.10**
**Doctor Communication**	**1.06**	**.06**	**.27**	**16.74**	**<.001**	**[.94, 1.19]**	**.05**
**Ambulance Arrival**	**.18**	**.07**	**.04**	**2.51**	**.012**	**[.04, .32]**	**.00**
**Care in 30 mins**	**.66**	**.06**	**.16**	**11.17**	**<.001**	**[.55, .78]**	**.02**
**New Meds**	**.21**	**.06**	**.05**	**3.47**	**<.001**	**[.09, .34]**	**.00**
**Had tests**	**.20**	**.07**	**.04**	**2.77**	**.006**	**[.06, .35]**	**.00**
Step 3 (SCQ)	b	SE	B	*t*	*p*	95% CI	*f*^2^
Constant	-2.74	.30		-9.14	<.001	[-3.33, -2.15]	
Age	.09	.01	.09	6.78	<.001	[.07, .12]	.01
Ethnicity	.06	.06	.01	.93	.356	[-.06, .18]	.00
Gender	.08	.05	.02	1.83	.067	[-.01, .17]	.00
Education	-.02	.01	-.02	-1.99	.047	[-.05, .00]	.00
Born in Canada	-.03	.06	-.01	-.47	.636	[-.16, .09]	.00
Financial Situation	-.02	.02	-.01	-1.20	.228	[-.06, .02]	.00
Health Today	.01	.00	.06	4.17	<.001	[.00, .01]	.00
Current Pain	-.12	.05	-.03	-2.34	.019	[-.22, -.02]	.00
EQ5D Pain	-.08	.03	-.04	-2.80	.005	[-.14, -.02]	.00
EQ5D Anxiety/Depression	.06	.03	.03	2.31	.021	[.01, .12]	.00
EQ5D Self Care	.07	.04	.02	1.58	.113	[-.02, .16]	.00
EQ5D Mobility	.02	.03	.01	.73	.463	[-.04, .09]	.00
EQ5D Usual Activities	-.01	.03	-.01	-.44	.659	[-.07, .04]	.00
Overall health	.01	.03	.01	.55	.581	[-.04, .07]	.00
Nurse Communication	.42	.05	.13	9.08	<.001	[.33, .51]	.01
Doctor Communication	.30	.05	.08	5.54	<.001	[.20, .41]	.00
Ambulance Arrival	.12	.06	.03	2.15	.031	[.01, .23]	.00
Care in 30 mins	.40	.05	.10	8.28	<.001	[.31, .50]	.01
New Meds	.13	.05	.03	2.68	.007	[.04, .23]	.00
Had tests	.09	.06	.02	1.48	.139	[-.03, .20]	.00
**SCQ**	**1.61**	**.04**	**.60**	**39.96**	**<.001**	**[1.53, 1.69]**	**.23**

*N* = 2998. Unstandardized effects denoted with b. Standardized effects denoted with B. Standard error denoted with SE. Cohen’s f squared (*f*^2^) represent effect sizes. Gender coded as men = 1, women and other gender groups = 2. Ethnicity coded as white/European =1, all other ethnicities = 2. Bolded text denotes new variables in the applicable step. Although the item “current pain” was positioned as part of the EDPEC, we felt it was best included under “patient information” rather than “patient experience” for the purposes of stepwise hierarchical multiple linear regression. For the purposes of the stepwise hierarchical multiple regression, we felt that the EQ-5D was best positioned with other demographic and patient information questions, as it addresses patients’ quality of life, rather than specific experiences at the ED. Several variables were not included in the multiple regressions, as they were only completed by a portion of participants, which would very strongly limit sample size for these analyses. Additionally, several of these variables were excluded from the multiple regressions as they were dependent on other included variables, and thus are not computable (i.e., the variables were constants). For example, patients were asked if they experienced any pain during their emergency department visit. If patients did experience pain, they were provided the question “during this emergency department visit, did the doctors and nurses try to help reduce your pain?”)

In step one, demographic and patient information variables statistically significantly contributed to the model, *F*(14, 2984) = 22.05, *p* < .001, *R*^*2*^ = .09. In step 2, patient experience variables statistically significantly added to the model, *F*(20, 2978) = 125.59, *p* < .001, *R*^*2*^ = .45 (*R*^*2*^ Δ = .36, *p* < .001). In step 3, the SCQ statistically significantly added to the model, *F*(21, 2977) = 259.80, *p* < .001, *R*^*2*^ = .65 (*R*^*2*^ Δ = .19, *p* < .001).

Adding the SCQ-ED to the regression model in step 3 explained an additional 19% variance in overall quality care ratings, *p* < .001. Overall, with the inclusion of the SCQ, we were able to explain 65% (versus 46%) of the variance in ED patients’ overall quality care ratings.

The full model significantly predicted overall quality care ratings, A total of 4 variables statistically predicted care ratings with effect sizes *f*^*2*^ ≥ .01. In order of effect size, these significant variables were the SCQ-ED (*f*^*2*^=.23), nurse communication (*f*^*2*^=.01), whether patients received care within 30 minutes (*f*^*2*^=.01), and age (*f*^*2*^=.01); see Table 4.

### Investigating demographic group differences in compassion

To assess whether patients reported experiences of compassion differed by demographic grouping, one-way ANOVAs were conducted. If statistically significant ANOVAs were identified, post-hoc analyses were utilized to better understand these differences. To supplement these analyses, a multivariate regression predicting SCQ-ED mean scores can be found in Appendix B.

### Ethnicity and compassion

A one-way ANOVA was conducted, indicating a statistically significant difference between ethnic groups was found, *F*(7, 4040) = 2.83, η^2^ = .01, *p* = .006. Tukey’s post-hoc pairwise comparisons revealed that compassion scores belonging to those that identified as Indigenous People of Canada were statistically significantly lower than those that identified as white (Mean Difference = -.17, SE = .05, 95% CI = [-.34, -.01], *p* = .033).

### Gender and compassion

A statistically significant difference between groups was found, *F*(4, 4455) = 11.58, η^2^ = .01, *p* <.001. Tukey’s post-hoc pairwise comparisons revealed that those that identified as women reported statistically significantly lower levels of compassion than those that identified as men (Mean Difference = -.15, SE = .02, 95% CI = [-.21, -.09], *p* <.001).

### Other variables and compassion

No statistically significant differences were found between groups by language spoken (*p* = .797), regional health zone (*p* = .059), education (*p* = .941), or reason for their ED visit (*p* = .345)

### Exploratory demographic group differences in overall care ratings

Given that mean compassion scores differed by ethnicity and gender, we opted exploratorily assess whether similar differences existed for these demographic groups by overall care ratings. As such, we conducted additional one-way ANOVAs for care ratings by ethnicity and gender.

### Ethnicity and care ratings

A statistically significant difference between ethnicity groups was found, *F*(7, 4032) = 5.49, η^2^ = .01, *p* <.001. Tukey’s post-hoc pairwise comparisons revealed that those that identified as Indigenous peoples of Canada reported statistically significantly lower overall care ratings (Mean Difference = -.47, SE = .14, 95% CI = [-.91, -.03], *p* = .027). They also reported statistically significantly lower care ratings than Southeast Asian patients (Mean Difference = -.71, SE = .21, 95% CI = [-1.35, -.07], *p* = .017) and Latin American/South American/Hispanic patients, (Mean Difference = -.84, SE = .25, 95% CI = [-1.61, -.07], *p* = .020). Additionally, other pairwise differences indicated that such that South Asian participants reported statistically significantly lower care ratings than white patients (Mean Difference = .53, SE = .15, 95% CI = [.09, -.97], *p* = .006.), Southeast Asian patients (Mean Difference = -.78, SE = .21, 95% CI = [-1.42, -.13], *p* = .006), and Latin American/South American/Hispanic patients, (Mean Difference = -.91, SE = .25, 95% CI = [-1.67, -.14], *p* = .008). Finally, Latin American/South American/Hispanic patients reported statistically significantly higher care ratings than patients with mixed ethnicities (Mean Difference = .97, SE = .31, 95% CI = [.02, 1.92], *p* = .042).

### Gender and care ratings

A statistically significant difference between gender groups was found, *F*(4, 4042) = 5.74, η^2^ = .01, *p* <.001. Tukey’s post-hoc pairwise comparisons revealed that women statistically significantly reported lower care ratings than men (Mean Difference = -.28, SE = .06, 95% CI = [-.45, -.11], *p* < .001.)

#### Demographic group differences in overall care ratings controlling for compassion

Results of the above ANOVAs indicated that the group in overall care ratings for Indigenous (*vs* white) patients, and women (*vs*. men) operated in the same direction as the differences found in compassion scores. To further explore whether patients’ reported compassion played a role in these discrepancies patients’ overall care ratings two Analyses of Covariance (ANCOVA) with mean SCQ-ED scores included as the covariate were explored and detailed below.

##### Ethnicity, compassion, and care ratings

A statistically significant relationship was found for mean SCQ-ED scores *F*(1, 4020) = 5535.01, *<*.001, and ethnicity *F*(7, 4020) = 3.36, *p* = .001. The model had a *R*^2^ = .58. This can be interpreted such that compassion explained a very large additional proportion of variance in overall care ratings, consistent with the multiple regression results above. Ethnicity remained a statistically significant predictor of overall care ratings however, but had a reduced *F* value, indicating that compassion may have explained some (but not all) of these differences in overall care ratings.

##### Gender, compassion, and care ratings

A statistically significant relationship was found for mean SCQ-ED scores *F*(1, 4028) = 6112.27, *<*.001 and overall care ratings. However, gender did not have a statistically significant relationship with overall care ratings *F*(4, 4028) = .20, *p* = .940. The model had a *R*^2^ = .58. This can be interpreted such that compassion explained a very large additional proportion of variance in overall care ratings, consistent with the multiple regression results above. Gender however became non-significant in the presence of mean SCQ-ED scores, indicating that the lower care ratings reported by women (*vs* men) were likely due to differences in perceived compassion in the ED.

## Discussion

To accomplish our main objective of understanding how compassion contributes to overall quality care ratings when considered within the broader landscape of existing patient experience measures, we used stepwise hierarchical linear multiple regression. We discovered that compassion, as measured by the SCQ-ED, was by far, the variable with the strongest prediction of overall quality care ratings, with a moderate-to-large effect size. Additionally, our analyses indicated that the inclusion of the SCQ-ED uniquely explained sizable (nearly 20%) additional variance in overall quality care ratings - the main outcome variable in the EDPEC, and other patient experience surveys including the Consumer Assessment of Healthcare Providers and Systems (CAHPS) surveys [23]. When one considers the salience of compassion to overall quality care ratings, above and beyond the existing domains of patient experience embedded within these questionnaires; the integration of compassion within these surveys and learning health systems [24] warrants serious consideration [25].

In addition to demonstrating that compassion was a key predictor of patients’ quality care ratings, another novel finding of this study was that receiving compassionate care varied according to patient demographic group (i.e., ethnicity and gender). First, we noted that women reported statistically significant lower levels of compassion and overall care ratings in the ED when compared to men. Indeed, follow-up analyses indicated that these differences in compassion, as measured by the SCQ-ED were very likely responsible for effects such that reported women lower overall care ratings than men. These quantifiable differences in compassion, as measured by the SCQ-ED are consistent with findings of discrimination in healthcare delivery for women versus men, which have found that personal experiences of prejudice are associated with reduced adherence to healthcare screening guidelines and pre-emptive health behaviours such as going to see a doctor when seriously ill [26]. Our findings that women experienced both less compassion and reported reduced overall care rating in ED settings merits further investigation within the context of experiences of prejudice and discrimination.

Additionally, we found statistically significant differences in the responses on the SCQ-ED between Indigenous and white/European patients. Specifically, we found that Indigenous patients reporting lower levels of compassion, as well as lower overall care ratings. This is consistent with findings that Indigenous peoples experience significant health disparities when compared to non-indigenous groups [21, 27]. Exploratory follow-up analyses (i.e., ANCOVA) examined group differences on overall care ratings, and indicated that the differences in patient-reported compassion may have played a role in the differences in overall care ratings reported by Indigenous patients. Our findings also echo recent work which found that First Nations individuals in Alberta were almost twice as likely to leave EDs without receiving care than non-First Nations patients, at least in part due to prejudice and discrimination. Follow-up interviews described First Nations’ patients facing anti-Indigenous stereotypes in diagnostic questions and case management, overhearing prejudicial attitudes being expressed by their providers, and discriminatory behaviours surrounding their quality of care [28]. We recommend future research, in partnership with Indigenous and diverse gender communities, and the deployment of improved EDI training in healthcare settings. Such approaches would help explore whether understandings and experiences of compassion differ, along with the development of studies investigating the role of how institutional racism, sexism, unconscious bias, and stigma operate in the inequitable access to and provision of compassion to different social groups [29, 30]. Further, we encourage that future research explore potentially interactive effects between patient and HCP sociodemographic features on patient-perceived compassion (e.g., do patients that receive care from providers with similar demographic identities, or cultural backgrounds feel they received more compassionate care?).

The validation of the SCQ-ED in this study also provides health leaders, researchers, and clinicians a valid and reliable measure to routinely assess patients experiences of compassion, and in doing so, an important, but previously hidden ingredient of quality care. We would like to highlight that the findings in this study are strengthened by our large sample size (N = 4501), and that our sample was derived from 14 EDs. Such a tool has potential utility for monitoring ED quality care, assessing interventions aimed at improving compassion, and providing patients with the ability to provide feedback on a central, but previously unmeasurable, aspect of the patient experience. We also note that a short 5-item SCQ measure is available (Sinclair et al, 2021), which has maintained the excellent reliability and validity of the full SCQ-ED. This short measure reduces patient burden and allows for greater flexibility where survey length is a concern. At a health systems level, the SCQ-ED could be considered a factor in hospital rating scores, while providing opportunities for health organizations to potentially establish and monitor benchmarks of compassion at a unit, institutional, regional or national level, and among various patient groups. Such benchmarking would allow for clear and tenable research connections between patient-reported compassion and a host of important clinical outcomes (e.g., patient recovery times, symptom severity, survival, etc.).

### Study limitations

This study is limited in its recall period, such that patients were asked to recall their most recent ED visit. As such, there is a level of variability in time from healthcare experienced to completion of the questionnaire, potentially limiting some specificity in recall. Additionally, we recognize several ethnic groups were not represented in our sample, specifically individuals identifying as Black or African American, and Middle Eastern, North African, and West African patients. We recognize this lack of full representation in the respondents and note that it is possible that such limited responding may have biased our results in an unknown direction. We therefore strongly recommend that future research include these populations. We also believe it is important to note that, our response rate may have been limited (i.e., 23%). Due to the data being collected as part of the provincial health authority’s standardized patient experience survey, we were limited to data they were authorized to collect and share. This did not include the demographic data of the initial population sampled.

It is important to acknowledge that although this study was conducted among a large sample of patients from a variety of regional and metropolitan EDs, our data was exclusively collected from the Canadian province of Alberta. We recommend the replication of these results in other jurisdictions, both nationally and internationally. While the initial results from ongoing studies (i.e., Spain, Italy, Portugal) validating the SCQ indicate that the SCQ functions well across cultures, future studies are required to assess the transferability of the SCQ-ED across cultures [31, 32]. Our very large sample provides us with an excellent opportunity to conduct analyses that can detect small and complex effects, without worrying about insufficient statistical power. While large sample sizes allow for the detection small and complex effects, it also comes with important caveats, including interpreting our findings in conjunction with our provided effect sizes and confidence intervals. Finally, data collection occurred during the COVID-19 pandemic and as such future studies outside the context of a pandemic are needed to verify and extend our results [33].

We acknowledge the limitations of cross-sectional studies, as such designs are insufficient for confirming etiological relationships between compassion and important outcome measures, such as overall patient experience. We recommend future studies employ both experimental and longitudinal designs to better establish causal relationships between perceptions of compassion in ED settings, quality care ratings and other important outcomes (e.g., recovery time, patient complaints, etc.).

In summary, this cross-sectional study of patient experience measures in EDs, we identified compassion as strongly associated with patients overall quality care ratings, presenting researchers with compelling evidence to consider the inclusion of compassion as a core domain of the patient experience. Finally, this study provided healthcare leaders, policy makers, and providers evidence that improving compassion is an essential and potent means for improving quality care, that can no longer be dismissed.

### Supplementary Information

Supplementary Material 1. 

## Data Availability

The data that support the findings of this study are not publicly available due to privacy restrictions. However, anonymized data may be available from the Health Quality Council of Alberta upon reasonable request. Please contact Dr. Markus Lahtinen (Markus.Lahtinen@hqca.ca) with requests.

## Abbreviations

ANOVA: Analysis of Variance
CAHPS: Consumer Assessment of Healthcare Providers and Systems
CFREB: Conjoint Faculties Research Ethics Board
ED: Emergency Department
EDPEC: Emergency Department Patient Experience of Care
HQCA: Health Quality Council of Alberta
MD: Mean difference
SCQ-ED: Sinclair Compassion Questionnaire – Emergency Department
STROBE: Strengthening the Reporting of Observational Studies in Epidemiology
