# Supplementary material for: What role does compassion have on quality care ratings? A regression analysis and validation of the SCQ in emergency department patients
Source: BMC Emerg Med. 2024 Jul 18;24:124. doi: 10.1186/s12873-024-01040-8 (PMC11264741; doi:10.1186/s12873-024-01040-8)
Supplement: Supplementary file 1 — Supplementary Material 1. [file 12873_2024_1040_MOESM1_ESM.docx]

**Online Supplementary Materials**

1. Appendix A. Health Quality Council of Alberta ED Questionnaire, Pages 2-9
2. Appendix B. Validity and Missing Values Analyses for SCQ-ED, Pages 10-16
   1. eFigure 1 Final Factor Loadings for Confirmatory Factor Analysis Model for the SCQ-ED, Page 11
   2. eFigure 2, Unspecified Confirmatory Factor Analysis Model for the SCQ-ED, Page 12
   3. eTable 1, Stepwise Multiple Regression Results for SCQ-ED, Page 14
3. Appendix C. SCQ Item Intercorrelations Page 16
4. Appendix D. Pearson and Point-Biserial Correlations, Page 17

**Appendix A. Health Quality Council of Alberta ED Questionnaire**

**GOING TO THE EMERGENCY DEPARTMENT**

1. Thinking about this visit, what was the main reason why you went to the emergency department?

- An accident or injury
- A new health problem NOT related to COVID-19
- A new health problem related to COVID-19
- An ongoing health condition or concern

1. Why did you choose to go to the emergency department, instead of somewhere else such as a doctor's office? FILL-IN ALL THAT APPLY

- The emergency department was the only choice available at the time.
- The emergency department was the most convenient place to go.
- I (we) thought the emergency department was the best place for my medical problem.
- I was told to go to the emergency department rather than somewhere else. → ***If “told to go”, ask Questions a) and b)***
- Other: ________________________
  1. Were you told to go to the emergency department by a nurse on the Health Link (8-1-1) phone line?
- Yes
- No
  1. Did you complete an online symptom checker, like the Alberta Health Services COVID-19 Self-Assessment, and it told you to call 9-1-1 or go to the nearest emergency department?
- Yes
- No

1. For this visit, did you go to the emergency department in an ambulance?

- Yes
- No

**DURING YOUR EMERGENCY**

**DEPARTMENT VISIT**

1. During this emergency department visit, did you get care within 30 minutes of getting to the emergency department?

- Yes
- No

1. During this emergency department visit, were you given any medicine that you had not taken before?

- Yes
- Don’t Know → ***If No, go to Question 7***
- No → ***If No, go to Question 7***

1. Before giving you any new medicine, did the doctors or nurses describe possible side effects to you in a way you could understand?

- Yes, definitely
- Yes, somewhat
- No

1. During this emergency department visit, did you have any pain?

- Yes
- No → ***If No, go to Question 11***

1. During this emergency department visit, did the doctors and nurses try to help reduce your pain?

- Yes, definitely
- Yes, somewhat
- No

1. During this emergency department visit, did you get medicine for pain?

- Yes
- No → ***If No, go to Question 11***

1. Before giving you pain medicine, did the doctors and nurses describe possible side effects in a way you could understand?

- Yes, definitely
- Yes, somewhat
- No

1. During this emergency department visit, did you have a blood test, x-ray, or any other test?

- Yes
- No → ***If No, go to Question 13***

1. During this emergency department visit, did doctors and nurses give you as much information as you wanted about the results of these tests?

- Yes, definitely
- Yes, somewhat
- No

**PEOPLE WHO TOOK CARE OF YOU** **IN THE EMERGENCY DEPARTMENT**

1. During this emergency department visit, how often did nurses introduce themselves to you?

- Never
- Sometimes
- Usually
- Always

1. During this emergency department visit, how often did nurses treat you with courtesy and respect?

- Never
- Sometimes
- Usually
- Always

1. During this emergency department visit, how often did nurses listen carefully to you?

- Never
- Sometimes
- Usually
- Always

1. During this emergency department visit, how often did nurses explain things in a way you could understand?

- Never
- Sometimes
- Usually
- Always

1. During this emergency department visit, how often did doctors introduce themselves to you?

- Never
- Sometimes
- Usually
- Always

1. During this emergency department visit, how often did doctors treat you with courtesy and respect?

- Never
- Sometimes
- Usually
- Always

1. During this emergency department visit, how often did doctors listen carefully to you?

- Never
- Sometimes
- Usually
- Always

1. During this emergency department visit, how often did doctors explain things in a way you could understand?

- Never
- Sometimes
- Usually
- Always

**OVERALL EXPERIENCE**

1. Using any number from 0 to 10, where 0 is the worst care possible and 10 is the best care possible, what number would you use to rate your care during this emergency department visit?

- 0 Worst care possible
- 1
- 2
- 3
- 4
- 5
- 6
- 7
- 8
- 9
- 10 Best care possible

**ABOUT YOU**

1. In general, how would you rate your overall health?

- Excellent
- Very good
- Good
- Fair
- Poor

1. EQ-5D

*(Note to interviewer: please read the following to the respondent)*

We are trying to find out what you think about your health. I will first ask you some simple questions about your health TODAY. I will then ask you to rate your health on a measuring scale. I will explain what to do as I go along but please interrupt me if you do not understand something or if things are not clear to you. Please also remember that there are no right or wrong answers. We are interested here only in your personal view.

**First I am going to read out some questions. Each question has a choice of five answers. Please tell me which answer best describes your health TODAY. Do not choose more than one answer in each group of questions.**

*(Note to interviewer: it may be necessary to remind the respondent regularly that the timeframe is TODAY. It may also be necessary to repeat the questions verbatim.)*

MOBILITY

First I'd like to ask you about mobility. Would you say that:

- ^1^ You have no problems in walking about?
- ^2^ You have slight problems in walking about?
- ^3^ You have moderate problems in walking about?
- ^4^ You have severe problems in walking about?
- ^5^ You are unable to walk about?

*(Note to interviewer: mark the appropriate box on the EQ-5D questionnaire)*

SELF-CARE

Next I'd like to ask you about self-care. Would you say that:

- ^1^ You have no problems washing or dressing yourself?
- ^2^ You have slight problems washing or dressing yourself?
- ^3^ You have moderate problems washing or dressing yourself?
- ^4^ You have severe problems washing or dressing yourself?
- ^5^ You are unable to wash or dress yourself?

*(Note to interviewer: mark the appropriate box on the EQ-5D questionnaire)*

USUAL ACTIVITIES

Next I'd like to ask you about your usual activities, for example work, study, housework, family or leisure activities. Would you say that:

- ^1^ You have no problems doing your usual activities?
- ^2^ You have slight problems doing your usual activities?
- ^3^ You have moderate problems doing your usual activities?
- ^4^ You have severe problems doing your usual activities?
- ^5^ You are unable to do your usual activities?

*(Note to interviewer: mark the appropriate box on the EQ-5D questionnaire)*

PAIN / DISCOMFORT

Next I'd like to ask you about pain or discomfort. Would you say that:

- ^1^ You have no pain or discomfort?
- ^2^ You have slight pain or discomfort?
- ^3^ You have moderate pain or discomfort?
- ^4^ You have severe pain or discomfort?
- ^5^ You have extreme pain or discomfort?

*(Note to interviewer: mark the appropriate box on the EQ-5D questionnaire)*

ANXIETY / DEPRESSION

Finally I'd like to ask you about anxiety or depression. Would you say that:

- ^1^ You are not anxious or depressed?
- ^2^ You are slightly anxious or depressed?
- ^3^ You are moderately anxious or depressed?
- ^4^ You are severely anxious or depressed?
- ^5^ You are extremely anxious or depressed?

*(Note to interviewer: mark the appropriate box on the EQ-5D questionnaire)*

1. EQ VAS

Now, I would like to ask you to say how good or bad your health is TODAY.

I'd like you to try to picture in your mind a scale that looks rather like a thermometer. Can you do that? The best health you can imagine is marked 100 (one hundred) at the top of the scale and the worst health you can imagine is marked 0 (zero) at the bottom.

EQ VAS: TASK

I would now like you to tell me the point on this scale where you would put your health today.

*(Note to interviewer: mark the scale at the point indicating the respondent’s ‘health today’)*

**DEMOGRAPHICS**

1. What is your age?

- 16 to 24
- 25 to 34
- 35 to 44
- 45 to 54
- 55 to 64
- 65 to 74
- 75 or older

1. Which of the following best represents your gender identity?

- Man
- Woman
- Non-binary
- Transgender
- I prefer to self-describe
  - Please tell us how you self-describe:_______________

1. What is the highest level of schooling that you have completed?

- No formal education
- Grade school or some high school
- Completed high school
- Post-secondary technical school (including Trade School)
- Some university or college
- Completed college diploma
- Completed university degree
- Post-grad degree (Masters or PhD)

1. What language do you mainly speak at home?

- English
- French
- Other:________________________

1. Were you born in Canada?

Yes

No

1. How many years have you lived in Canada?

_________________

1. People living in Canada come from many different cultural and racial backgrounds. Do you identify as a member of any of the following ethnic/racial groups? Please select any that apply

- White/European
- Indigenous Peoples of Canada (e.g., First Nations, Inuit, Metis)
- East Asian (e.g., Korean, Chinese, Japanese, Mongolian)
- South Asian (e.g., Indian, Pakistani, Sri Lankan)
- Southeast Asian (e.g., Filipino, Malaysian, Indonesian, Thai, Vietnamese, Laotian)
- Latin American/South American/Hispanic
- Black/African American
- Middle Eastern, North African, and West Asian (e.g., Iranian, Iraqi, Egyptian, Saudi, Syrian, Turkish, Yemeni).
- Other: ________________________

1. Which one of the following categories best describes the total annual income, before taxes, of all members of your household?

- Less than $25,000
- $25,000 to just under $50,000
- $50,000 to just under $75,000
- $75,000 to just under $100,000
- $100,000 to just under $150,000
- $150,000 to just under $200,000
- $200,000 or more

1. Which of the following best describes your financial situation?

- Very comfortable
- Comfortable
- Modestly comfortable
- Tight
- Very tight
- Poor

**Appendix B. Validity and Missing Values Analyses for SCQ-ED**

**Missing Values Analysis for the SCQ-ED**

The quantity of missing data (4197/4501 or 6.8%) for the SCQ-ED, as such, we conducted a missing value analysis. In these results, missing values per individual SCQ-ED question ranged between .7% to 3.1%. Little’s MCAR test was conducted using expectation maximization (Chi-Square 2060.81, DF 1215, *p* <.001). This indicates that the missing values may not be missing completely at random, however we note that Little’s MCAR test relies on chi-square tests. Chi-square tests are quite sensitive to sample size, and in large samples even quite small relationships may appear as statistically significant, even if not meaningful. As such, we also descriptively explored the summary of estimated means between all values and EM values. In total 10/15 SCQ-ED items had no differences in their estimated means, and the 5 items with differences did not exceed mean differences of .01 (on a 5-point Likert scale). When comparing the summary of estimated standard deviations between all values and EM estimates, any individual difference between item standard deviations did not exceed .003. As such, we believe it is highly unlikely that the exclusion of these missing values has had any meaningful impact on our CFA results.

**Confirmatory Factor Analysis for the SCQ-ED**

***CFA Criteria***

In order to confirm the factor structure of the SCQ uncovered in Sinclair and colleagues[1], confirmatory factor analysis (CFA) was employed using maximum likelihood estimation, with missing data excluded. We explored factor loadings and several common global fit indicators including model *χ^2^*, comparative fit index (CFI), standardized root-mean squared residual (SRMR), and root-mean-squared residuals (RMSEA). Fit criteria indicative of good fit includes non-significant *χ^2^* tests, CFI values above 0.95, SRMR values less than .08,[2-3] and RMSEA values below .08.[4].

***CFA Results***

A single latent factor model of compassion was selected for the CFA, consistent with previous factor analysis findings for other populations.[1] Only patients with complete data were included (N = 4197). Initial model estimation revealed excellent standardized factor loadings for all 15-items, ranging between .82 and .92. All global fit indices revealed adequate to good fit, with the exception of *χ^2^* being statistically significant, as well as RMSEA exceeding .08 (*χ^2^* = 3158.38, *p* <.001, CFI = .96, RMSEA = .09, SRMR = .02), see Appendix B for the unspecified model (i.e., model without residual covariances). Whereas global measures of fit are useful for assess the model as a whole, they do not necessarily identify specific sources of misspecification.[5] Thus, we examined local fit, determining that there were residual covariances between SCQ items outside of the single factor compassion model,[5-6] as was found by Sinclair and colleagues.[1] The model was then sequentially respecified by adding covariances between pairs of error terms with residual covariances exceeding 1.96, one-by-one (starting with the largest) until significant standardized residual covariances were no longer observed. This resulted in adding 9 residual associations to the model (see Figure 1A for final CFA, and Figure 2A for the unspecified model). Standardized factor loadings for this final respecified model remained very strong, ranging between .81 and .92, and global fit indices were improved (*χ^2^* = 1260.89, *p* <.001, CFI = .98, RMSEA = .06, SRMR = .01).

eFigure 1. Final Factor Loadings for Confirmatory Factor Analysis Model for the SCQ-ED


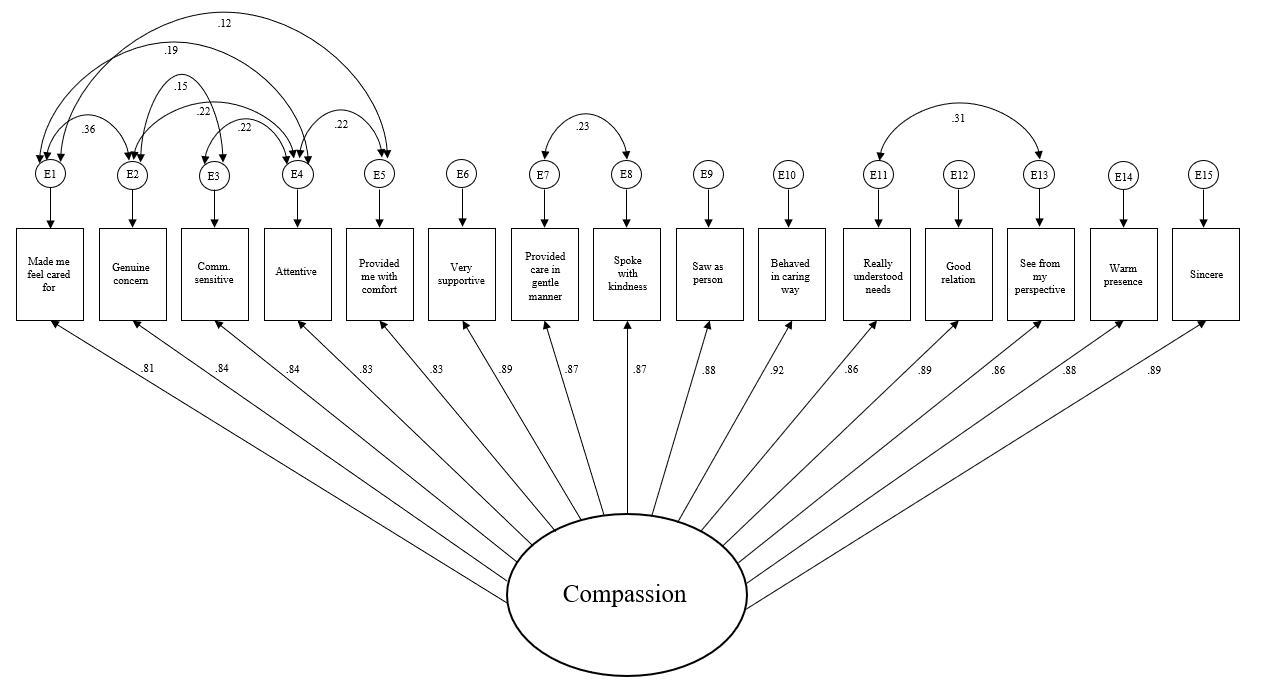


Note. Confirmatory factor analysis for the Sinclair Compassion Questionnaire. N = 4197. Standardized factor loadings reported. Fit indices: χ2 = 1260.89, p <.001, CFI = .98, RMSEA = .06, SRMR = .01. Cronbach’s alpha =.98. Es denote residuals.)

eFigure 2. Unspecified Confirmatory Factor Analysis Model for the SCQ-ED


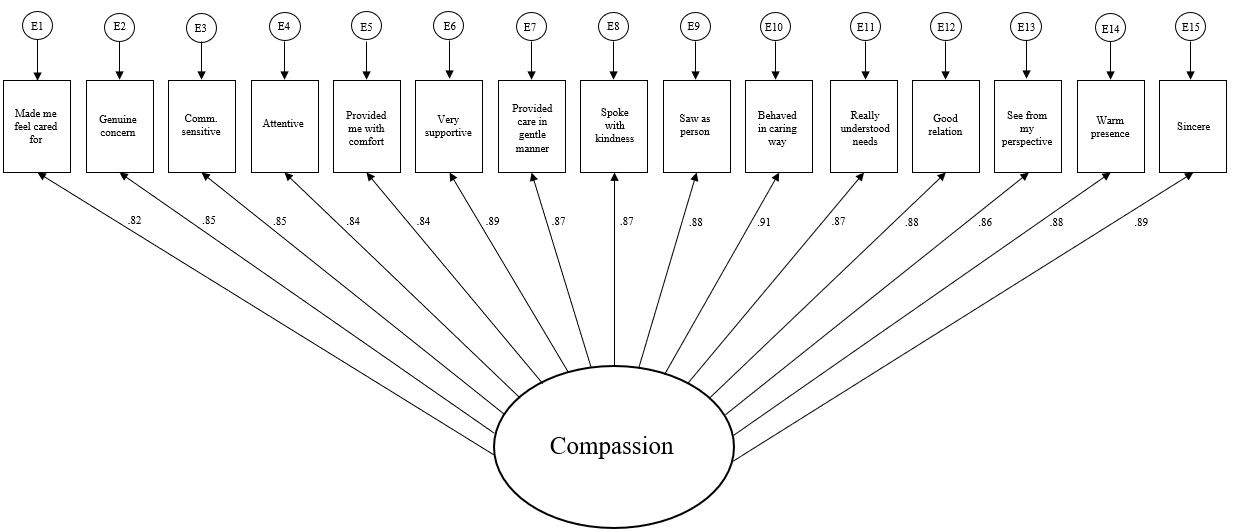


Note. Confirmatory factor analysis for the Sinclair Compassion Questionnaire. N = 4197. Standardized factor loadings reported. Fit indices: χ2 = 3158.38, p <.001, CFI = .96, RMSEA = .09, SRMR = .02. Cronbach’s alpha =3 .98. Es denote residuals

**Stepwise Hierarchical Linear Multiple Regression for the SCQ-ED**

A two-step hierarchical linear regression was conducted with the SCQ as the outcome variable. In the Step 1 the SCQ was regressed on demographic and patient information variables. In Step 2, variables relating to patient experience during their ED visit were added.

***Outcome: Sinclair Compassion Questionnaire (SCQ)***

A two-step hierarchical multiple regression was run to estimate scores on the SCQ from applicable continuous and dichotomously coded variables (*N* = 3005). In step one, demographic and patient information variables were added to the model, and statistically significantly added to the model estimates, *F*(14, 2991) = 18.49, *p* < .001, *R^2^* = .08. In Step 2, patient experience measures were added, and significantly added to the model, *F*(20, 2985) = 137.15, *p* <.001, *R^2^* = .48 (*R^2^* Δ = .40, *p* < .001).

A total of 3 variables statistically significantly estimated compassion ratings with effect sizes *f^2^* ≥ .01. In order of effect size, these significant variables were nurse communication, doctor communication, whether patients received care within 30 minutes, see Table 1A.

There were additional variables that significantly estimated the SCQ-ED but their effect sizes were very small (e.g., *f^2^* < .01), suggesting the need to exercise caution in interpretation. Large datasets such as ours provide valuable opportunities for data analysis, but they also can create challenges in interpreting statistical significance, through deflation of p-values (see Lin and colleagues [7] for commentary). We consider results meaningful only when they are statistically significant and demonstrate an adequate effect size.

eTable 1. Stepwise Multiple Regression Results for SCQ-ED

| Step 1 (Demographics) | b | SE | B | *t* | *p* | CI | *f*^2^ |
| --- | --- | --- | --- | --- | --- | --- | --- |
| Constant | 3.80 | .15 |  | 26.04 | <.001 | [3.51, 4.08] |  |
| Age | .04 | .01 | .11 | 5.40 | <.001 | [0.03, 0.06] | .01 |
| Ethnicity | .01 | .04 | .01 | .37 | .711 | [-0.06, 0.09] | .00 |
| Gender | -.12 | .03 | -.08 | -4.57 | <.001 | [-0.18, -0.07] | .01 |
| Education | -.01 | .01 | -.02 | -1.03 | .302 | [-0.02, 0.01] | .00 |
| Born in Canada | .06 | .04 | .03 | 1.45 | .146 | [-0.02, 0.13] | .00 |
| Financial Situation | .02 | .01 | .03 | 1.48 | .140 | [-0.01, 0.04] | .00 |
| Health Today | .00 | .00 | .12 | 4.64 | <.001 | [0.00, 0.01] | .01 |
| Current Pain | -.12 | .03 | -.07 | -3.99 | <.001 | [-0.18, -0.06] | .00 |
| EQ5D Pain | -.02 | .02 | -.03 | -1.15 | .249 | [-0.06, 0.01] | .00 |
| EQ5D Anxiety/Depression | -.06 | .02 | -.07 | -3.48 | <.001 | [-0.09, -0.02] | .00 |
| EQ5D Self Care | -.04 | .03 | -.03 | -1.42 | .156 | [-0.09, 0.01] | .00 |
| EQ5D Mobility | .02 | .02 | .03 | 1.04 | .298 | [-0.02, 0.06] | .00 |
| EQ5D Usual Activities | .02 | .02 | .03 | 1.12 | .262 | [-0.01, 0.05] | .00 |
| Overall health | .06 | .02 | .09 | 4.10 | <.001 | [0.03, 0.10] | .01 |
| Step 2 (Experience) | b | SE | B | *t* | *p* | CI | *f*^2^ |
| Constant | .32 | .14 |  | 2.39 | .017 | [0.06, 0.59] |  |
| Age | .02 | .01 | .05 | 3.37 | .001 | [0.01, 0.03] | .00 |
| Ethnicity | .05 | .03 | .03 | 1.63 | .103 | [-0.01, 0.10] | .00 |
| Gender | -.04 | .02 | -.03 | -1.92 | .055 | [-0.08, 0.00] | .00 |
| Education | -.01 | .01 | -.02 | -1.61 | .107 | [-0.02, 0.00] | .00 |
| Born in Canada | .01 | .03 | .01 | .39 | .700 | [-0.05, 0.07] | .00 |
| Financial Situation | .01 | .01 | .01 | .85 | .395 | [-0.01, 0.03] | .00 |
| Health Today | .00 | .00 | .06 | 3.08 | .002 | [0.00, 0.00] | .00 |
| Current Pain | -.06 | .02 | -.04 | -2.72 | .007 | [-0.11, -0.02] | .00 |
| EQ5D Pain | .00 | .01 | .00 | .17 | .862 | [-0.02, 0.03] | .00 |
| EQ5D Anxiety/Depression | -.02 | .01 | -.03 | -1.86 | .063 | [-0.05, 0.00] | .00 |
| EQ5D Self Care | -.01 | .02 | -.01 | -.69 | .493 | [-0.05, 0.03] | .00 |
| EQ5D Mobility | .01 | .02 | .02 | .96 | .337 | [-0.02, 0.05] | .00 |
| EQ5D Usual Activities | .02 | .01 | .02 | 1.20 | .229 | [-0.01, 0.04] | .00 |
| Overall health | .04 | .01 | .05 | 3.05 | .002 | [0.01, 0.06] | .00 |
| **Nurse Communication** | **.46** | **.02** | **.39** | **24.34** | **<.001** | **[0.43, 0.50]** | **.12** |
| **Doctor Communication** | **.47** | **.02** | **.32** | **20.25** | **<.001** | **[0.43, 0.52]** | **.08** |
| **Ambulance Arrival** | **.04** | **.03** | **.02** | **1.37** | **.171** | **[-0.02, 0.09]** | **.00** |
| **Care in 30 mins** | **.16** | **.02** | **.11** | **7.48** | **<.001** | **[0.12, 0.21]** | **.01** |
| **New Meds** | **.05** | **.02** | **.03** | **2.23** | **.026** | **[0.01, 0.10]** | **.00** |
| **Had tests** | **.07** | **.03** | **.04** | **2.64** | **.008** | **[0.02, 0.12]** | **.00** |

Note: N = 3005. Unstandardized effects denoted with b. Standardized effects denoted with B. Standard error denoted with SE. Cohen’s f squared (*f*^2^) represent effect sizes. B = Standardized coefficients. b = Unstandardized coefficients. Gender coded as men= 1, women and other gender groups = 2. Ethnicity coded as white/European =1, all other ethnicities = 2. Bolded text denotes new variables in the applicable step.

References

1. Sinclair S, Hack TF, MacInnis CC The COMPASS Research Team, et al. Development and validation of a patient-reported measure of compassion in healthcare: the Sinclair Compassion Questionnaire (SCQ) BMJ Open 2021;11:e045988.
2. Kline RB. Methodology in the social sciences. In: Principles and practice of structural equation modeling. 3^rd^ ed. New York: Guilford Press; 2011.
3. Hu LT, Bentler PM. Cutoff criteria for fit indexes in covariance structure analysis: Conventional criteria versus new alternatives. Structural equation modeling: a multidisciplinary journal. 1999 Jan 1;6(1):1-55.
4. MacCallum RC, Browne MW, Sugawara HM. Power analysis and determination of sample size for covariance structure modeling. Psychol Methods. 1996 Jun;1(2):130.
5. Padgett RN. Assessing local fit by approximating probabilities [Internet]. 2021 Feb 3 [cited 2023 Aug 3]. Available from: <https://scholarworks.uttyler.edu/sera2021/conference/evaluation/7/>
6. Maydeu-Olivares A, Shi D. Effect sizes of model misfit in structural equation models. Methodology. 2017 Jun;13(1):23-30.
7. Lin M, Lucas Jr HC, Shmueli G. Research commentary—too big to fail: large samples and the p-value problem. Information Systems Research. 2013 Dec;24(4):906-17

|  | | Made me feel cared for | Genuine Concern | Communicated sensitive | Attentive | Provided me with comfort | Very supportive | Provided care in gentle manner | Spoke with kindness | Saw as person | Behaved in caring way | Really understood needs | Good relationship | See from my perspective | Warm presence | Sincere |
| --- | --- | --- | --- | --- | --- | --- | --- | --- | --- | --- | --- | --- | --- | --- | --- | --- |
| Made me feel cared for | *r* | -- |  |  |  |  |  |  |  |  |  |  |  |  |  |  |
|  | *N* | 4462 |  |  |  |  |  |  |  |  |  |  |  |  |  |  |
| Genuine Concern | *r* | 0.79 | -- |  |  |  |  |  |  |  |  |  |  |  |  |  |
|  | *p* | <.001 |  |  |  |  |  |  |  |  |  |  |  |  |  |  |
|  | *N* | 4445 | 4466 |  |  |  |  |  |  |  |  |  |  |  |  |  |
| Communicated sensitive | *r* | 0.70 | 0.75 | -- |  |  |  |  |  |  |  |  |  |  |  |  |
|  | *p* | <.001 | <.001 |  |  |  |  |  |  |  |  |  |  |  |  |  |
|  | *N* | 4443 | 4450 | 4465 |  |  |  |  |  |  |  |  |  |  |  |  |
| Attentive | *r* | 0.74 | 0.77 | 0.76 | -- |  |  |  |  |  |  |  |  |  |  |  |
|  | *p* | <.001 | <.001 | <.001 |  |  |  |  |  |  |  |  |  |  |  |  |
|  | *N* | 4439 | 4442 | 4442 | 4459 |  |  |  |  |  |  |  |  |  |  |  |
| Provided me with comfort | *r* | 0.71 | 0.73 | 0.71 | 0.76 | -- |  |  |  |  |  |  |  |  |  |  |
|  | *p* | <.001 | <.001 | <.001 | <.001 |  |  |  |  |  |  |  |  |  |  |  |
|  | *N* | 4435 | 4435 | 4435 | 4430 | 4451 |  |  |  |  |  |  |  |  |  |  |
| Very supportive | *r* | 0.72 | 0.76 | 0.75 | 0.74 | 0.77 | -- |  |  |  |  |  |  |  |  |  |
|  | *p* | <.001 | <.001 | <.001 | <.001 | <.001 |  |  |  |  |  |  |  |  |  |  |
|  | *N* | 4440 | 4443 | 4446 | 4439 | 4437 | 4460 |  |  |  |  |  |  |  |  |  |
| Provided care in gentle manner | *r* | 0.70 | 0.72 | 0.74 | 0.72 | 0.72 | 0.79 | -- |  |  |  |  |  |  |  |  |
|  | *p* | <.001 | <.001 | <.001 | <.001 | <.001 | <.001 |  |  |  |  |  |  |  |  |  |
|  | *N* | 4437 | 4441 | 4441 | 4438 | 4432 | 4441 | 4458 |  |  |  |  |  |  |  |  |
| Spoke with kindness | *r* | 0.68 | 0.71 | 0.76 | 0.71 | 0.70 | 0.79 | 0.81 | -- |  |  |  |  |  |  |  |
|  | *p* | <.001 | <.001 | <.001 | <.001 | <.001 | <.001 | <.001 |  |  |  |  |  |  |  |  |
|  | *N* | 4447 | 4453 | 4450 | 4449 | 4440 | 4448 | 4449 | 4468 |  |  |  |  |  |  |  |
| Saw as person | *r* | 0.70 | 0.74 | 0.72 | 0.72 | 0.73 | 0.77 | 0.75 | 0.76 | -- |  |  |  |  |  |  |
|  | *p* | <.001 | <.001 | <.001 | <.001 | <.001 | <.001 | <.001 | <.001 |  |  |  |  |  |  |  |
|  | *N* | 4400 | 4404 | 4403 | 4399 | 4395 | 4400 | 4401 | 4411 | 4416 |  |  |  |  |  |  |
| Behaved in caring way | *r* | 0.73 | 0.76 | 0.77 | 0.75 | 0.74 | 0.81 | 0.80 | 0.83 | 0.82 | -- |  |  |  |  |  |
|  | *p* | <.001 | <.001 | <.001 | <.001 | <.001 | <.001 | <.001 | <.001 | <.001 |  |  |  |  |  |  |
|  | *N* | 4449 | 4452 | 4451 | 4448 | 4442 | 4450 | 4449 | 4459 | 4412 | 4468 |  |  |  |  |  |
| Really understood needs | *r* | 0.71 | 0.73 | 0.71 | 0.73 | 0.73 | 0.77 | 0.73 | 0.73 | 0.78 | 0.78 | -- |  |  |  |  |
|  | *p* | <.001 | <.001 | <.001 | <.001 | <.001 | <.001 | <.001 | <.001 | <.001 | <.001 |  |  |  |  |  |
|  | *N* | 4428 | 4430 | 4428 | 4427 | 4420 | 4429 | 4430 | 4439 | 4395 | 4441 | 4446 |  |  |  |  |
| Good relationship | *r* | 0.70 | 0.73 | 0.72 | 0.73 | 0.73 | 0.78 | 0.76 | 0.75 | 0.78 | 0.80 | 0.79 | -- |  |  |  |
|  | *p* | <.001 | <.001 | <.001 | <.001 | <.001 | <.001 | <.001 | <.001 | <.001 | <.001 | <.001 |  |  |  |  |
|  | *N* | 4415 | 4417 | 4416 | 4412 | 4409 | 4414 | 4414 | 4425 | 4382 | 4428 | 4411 | 4432 |  |  |  |
| See from my perspective | *r* | 0.70 | 0.72 | 0.71 | 0.71 | 0.72 | 0.76 | 0.72 | 0.71 | 0.77 | 0.77 | 0.82 | 0.80 | -- |  |  |
|  | *p* | <.001 | <.001 | <.001 | <.001 | <.001 | <.001 | <.001 | <.001 | <.001 | <.001 | <.001 | <.001 |  |  |  |
|  | *N* | 4349 | 4348 | 4348 | 4345 | 4344 | 4350 | 4347 | 4355 | 4325 | 4358 | 4347 | 4336 | 4363 |  |  |
| Warm presence | *r* | 0.69 | 0.71 | 0.73 | 0.72 | 0.72 | 0.77 | 0.75 | 0.78 | 0.78 | 0.80 | 0.75 | 0.79 | 0.77 | -- |  |
|  | *p* | <.001 | <.001 | <.001 | <.001 | <.001 | <.001 | <.001 | <.001 | <.001 | <.001 | <.001 | <.001 | <.001 |  |  |
|  | *N* | 4414 | 4416 | 4416 | 4413 | 4407 | 4416 | 4416 | 4424 | 4384 | 4428 | 4410 | 4399 | 4338 | 4431 |  |
| Sincere | *r* | 0.71 | 0.74 | 0.74 | 0.73 | 0.71 | 0.78 | 0.76 | 0.80 | 0.77 | 0.82 | 0.77 | 0.78 | 0.76 | 0.81 | -- |
|  | *p* | <.001 | <.001 | <.001 | <.001 | <.001 | <.001 | <.001 | <.001 | <.001 | <.001 | <.001 | <.001 | <.001 | <.001 |  |
|  | *N* | 4417 | 4419 | 4419 | 4418 | 4410 | 4416 | 4418 | 4426 | 4388 | 4431 | 4414 | 4404 | 4340 | 4403 | 4434 |

**Appendix C. SCQ Item Intercorrelations**

Notes: *r* denotes Pearson correlation coefficient.

| **Appendix D. Pearson and Point-Biserial Correlations** | | | | | | | | | | | | | | | | | | | | | | | | | | | | |
| --- | --- | --- | --- | --- | --- | --- | --- | --- | --- | --- | --- | --- | --- | --- | --- | --- | --- | --- | --- | --- | --- | --- | --- | --- | --- | --- | --- | --- |
|  | | SCQ | Care Rating | Nurse Comm | Doc Comm | Care in 30 Mins | Had Pain | Tried to Reduce Pain | Pain Meds | Pain Med Side Eff Discussed | New Meds | New Meds Discussed | Tests Comp | Test Info Discussed | Ambulance Arrival | Health Today | Overall Health | EQ5D Pain | EQ5D Mobility | EQ5D Usual Activities | EQ5D Self Care | EQ5D Anxiety/ Depression | Ethnicity | Gender | Education | Age | Financial Situation | Born in Canada |
| SCQ | *r* | 1 |  |  |  |  |  |  |  |  |  |  |  |  |  |  |  |  |  |  |  |  |  |  |  |  |  |  |
|  | *N* | 4485 |  |  |  |  |  |  |  |  |  |  |  |  |  |  |  |  |  |  |  |  |  |  |  |  |  |  |
| Care Rating | *r* | .76^***^ | 1 |  |  |  |  |  |  |  |  |  |  |  |  |  |  |  |  |  |  |  |  |  |  |  |  |  |
|  | *N* | 4455 | 4468 |  |  |  |  |  |  |  |  |  |  |  |  |  |  |  |  |  |  |  |  |  |  |  |  |  |
| Nurse Comm | *r* | .605^***^ | .57^***^ | 1 |  |  |  |  |  |  |  |  |  |  |  |  |  |  |  |  |  |  |  |  |  |  |  |  |
|  | *N* | 4432 | 4412 | 4442 |  |  |  |  |  |  |  |  |  |  |  |  |  |  |  |  |  |  |  |  |  |  |  |  |
| Doc Comm | *r* | .56^***^ | .51^***^ | .54^***^ | 1 |  |  |  |  |  |  |  |  |  |  |  |  |  |  |  |  |  |  |  |  |  |  |  |
|  | *N* | 4367 | 4349 | 4341 | 4376 |  |  |  |  |  |  |  |  |  |  |  |  |  |  |  |  |  |  |  |  |  |  |  |
| Care in 30 Mins | *r* | .26^***^ | .32^***^ | .21^***^ | .15^***^ | 1 |  |  |  |  |  |  |  |  |  |  |  |  |  |  |  |  |  |  |  |  |  |  |
|  | *N* | 4349 | 4330 | 4308 | 4246 | 4362 |  |  |  |  |  |  |  |  |  |  |  |  |  |  |  |  |  |  |  |  |  |  |
| Had Pain | *r* | -.08^***^ | -.10^***^ | -.07^***^ | -.07^***^ | -.10^***^ | 1 |  |  |  |  |  |  |  |  |  |  |  |  |  |  |  |  |  |  |  |  |  |
|  | *N* | 4447 | 4430 | 4404 | 4338 | 4333 | 4462 |  |  |  |  |  |  |  |  |  |  |  |  |  |  |  |  |  |  |  |  |  |
| Tried to Reduce Pain | *r* | .33^***^ | .35^***^ | .27^***^ | .22^***^ | .24^***^ | .^c^ | 1 |  |  |  |  |  |  |  |  |  |  |  |  |  |  |  |  |  |  |  |  |
|  | *N* | 3056 | 3042 | 3027 | 3007 | 2981 | 3050 | 3067 |  |  |  |  |  |  |  |  |  |  |  |  |  |  |  |  |  |  |  |  |
| Pain Meds | *r* | .17^***^ | .18^***^ | .15^***^ | .11^***^ | .11^***^ | .^c^ | .55^***^ | 1 |  |  |  |  |  |  |  |  |  |  |  |  |  |  |  |  |  |  |  |
|  | *N* | 3029 | 3014 | 3000 | 2980 | 2953 | 3019 | 3005 | 3039 |  |  |  |  |  |  |  |  |  |  |  |  |  |  |  |  |  |  |  |
| Pain Med Side Eff Discussed | *r* | .22^***^ | .21^***^ | .23^***^ | .18^***^ | .07^**^ | .^c^ | .18^***^ | .^c^ | 1 |  |  |  |  |  |  |  |  |  |  |  |  |  |  |  |  |  |  |
|  | *N* | 1677 | 1668 | 1671 | 1652 | 1639 | 1676 | 1672 | 1661 | 1683 |  |  |  |  |  |  |  |  |  |  |  |  |  |  |  |  |  |  |
| New Meds | *r* | .07^***^ | .08^***^ | .05^**^ | .03^*^ | .08^***^ | .06^***^ | .26^***^ | .30^***^ | 0.03 | 1 |  |  |  |  |  |  |  |  |  |  |  |  |  |  |  |  |  |
|  | *N* | 4300 | 4282 | 4259 | 4195 | 4197 | 4282 | 2944 | 2927 | 1601 | 4313 |  |  |  |  |  |  |  |  |  |  |  |  |  |  |  |  |  |
| New Meds Discussed | *r* | .26^***^ | .21^***^ | .24^***^ | .22^***^ | .08^**^ | -0.039 | .18^***^ | 0.03 | .69^***^ | .^c^ | 1 |  |  |  |  |  |  |  |  |  |  |  |  |  |  |  |  |
|  | *N* | 1143 | 1145 | 1143 | 1131 | 1127 | 1142 | 834 | 837 | 647 | 1149 | 1149 |  |  |  |  |  |  |  |  |  |  |  |  |  |  |  |  |
| Test Comp | *r* | .10^***^ | .11^***^ | .08^***^ | .05^**^ | .07^***^ | .11^***^ | .11^***^ | .13^***^ | 0.02 | .09^***^ | 0.04 | 1 |  |  |  |  |  |  |  |  |  |  |  |  |  |  |  |
|  | *N* | 4424 | 4406 | 4382 | 4318 | 4307 | 4407 | 3032 | 3006 | 1662 | 4263 | 1139 | 4439 |  |  |  |  |  |  |  |  |  |  |  |  |  |  |  |
| Test Info Discussed | *r* | .38^***^ | .37^***^ | .33^***^ | .41^***^ | .15^***^ | -.04^*^ | .22^***^ | .10^***^ | .20^***^ | 0.00 | .26^***^ | .^c^ | 1 |  |  |  |  |  |  |  |  |  |  |  |  |  |  |
|  | *N* | 3568 | 3555 | 3541 | 3505 | 3468 | 3555 | 2533 | 2510 | 1458 | 3418 | 983 | 3561 | 3580 |  |  |  |  |  |  |  |  |  |  |  |  |  |  |
| Ambulance Arrival | *r* | .03^*^ | .09^***^ | 0.00 | -.04^**^ | .25^***^ | -.07^***^ | .15^***^ | .17^***^ | -.07^**^ | .07^***^ | -0.04 | .09^***^ | -.04^*^ | 1 |  |  |  |  |  |  |  |  |  |  |  |  |  |
|  | *N* | 4471 | 4454 | 4429 | 4363 | 4351 | 4453 | 3061 | 3031 | 1677 | 4299 | 1149 | 4426 | 3570 | 4487 |  |  |  |  |  |  |  |  |  |  |  |  |  |
| Health Today | *r* | .15^***^ | .16^***^ | .15^***^ | .14^***^ | -0.02 | -0.008 | 0.01 | -0.02 | .09^***^ | -0.01 | 0.05 | -0.02 | .07^***^ | -.19^***^ | 1 |  |  |  |  |  |  |  |  |  |  |  |  |
|  | *N* | 4259 | 4255 | 4224 | 4162 | 4142 | 4239 | 2902 | 2875 | 1590 | 4095 | 1104 | 4215 | 3407 | 4260 | 4273 |  |  |  |  |  |  |  |  |  |  |  |  |
| Health Overall | *r* | .18^***^ | .16^***^ | .16^***^ | .16^***^ | -0.02 | 0.01 | .04^*^ | -0.02 | .11^***^ | 0.03 | 0.06 | -0.03 | .10^***^ | -.17^***^ | .59^***^ | 1 |  |  |  |  |  |  |  |  |  |  |  |
|  | *N* | 4418 | 4410 | 4376 | 4313 | 4298 | 4395 | 3027 | 2998 | 1654 | 4246 | 1134 | 4371 | 3526 | 4420 | 4243 | 4433 |  |  |  |  |  |  |  |  |  |  |  |
| EQ5D Pain | *r* | -.11^***^ | -.12^***^ | -.13^***^ | -.13^***^ | 0.03 | .16^***^ | -0.03 | .04^*^ | -.08^**^ | 0.01 | -0.05 | .04^*^ | -.09^***^ | .15^***^ | -.48^***^ | -.38^***^ | 1 |  |  |  |  |  |  |  |  |  |  |
|  | *N* | 4436 | 4425 | 4394 | 4330 | 4315 | 4412 | 3029 | 3003 | 1665 | 4267 | 1143 | 4389 | 3544 | 4436 | 4264 | 4396 | 4450 |  |  |  |  |  |  |  |  |  |  |
| EQ5D Mobility | *r* | -.06^***^ | -.04^**^ | -.07^***^ | -.09^***^ | .06^***^ | 0.02 | -0.00 | 0.02 | -.10^***^ | -0.02 | -0.05 | .04^*^ | -.03^*^ | .24^***^ | -.46^***^ | -.37^***^ | .50^***^ | 1 |  |  |  |  |  |  |  |  |  |
|  | *N* | 4436 | 4426 | 4394 | 4331 | 4315 | 4413 | 3027 | 3000 | 1665 | 4266 | 1140 | 4389 | 3543 | 4437 | 4262 | 4398 | 4435 | 4451 |  |  |  |  |  |  |  |  |  |
| EQ5D Usual Activities | *r* | -.08^***^ | -.08^***^ | -.10^***^ | -.10^***^ | .03^*^ | .05^**^ | -0.02 | .04^*^ | -.08^**^ | 0.01 | -0.06 | .07^***^ | -.06^***^ | .20^***^ | -.50^***^ | -.38^***^ | .52^***^ | .62^***^ | 1 |  |  |  |  |  |  |  |  |
|  | *N* | 4422 | 4411 | 4380 | 4316 | 4300 | 4398 | 3022 | 2996 | 1663 | 4255 | 1139 | 4375 | 3531 | 4423 | 4248 | 4385 | 4425 | 4425 | 4437 |  |  |  |  |  |  |  |  |
| EQ5D Self Care | *r* | -.06^***^ | -.05^**^ | -.07^***^ | -.07^***^ | 0.03 | 0.03 | -0.02 | 0.01 | -0.04 | 0.00 | -.10^**^ | 0.02 | -0.03 | .19^***^ | -.34^***^ | -.26^***^ | .35^***^ | .51^***^ | .51^***^ | 1 |  |  |  |  |  |  |  |
|  | *N* | 4443 | 4432 | 4401 | 4337 | 4320 | 4420 | 3031 | 3004 | 1666 | 4274 | 1143 | 4397 | 3549 | 4444 | 4266 | 4403 | 4444 | 4444 | 4432 | 4458 |  |  |  |  |  |  |  |
| EQ5D Anxiety/Depression | *r* | -.16^***^ | -.14^***^ | -.15^***^ | -.15^***^ | -0.02 | .06^***^ | -0.03 | 0.03 | -.06^*^ | 0.02 | -.08^**^ | 0.01 | -.09^***^ | .08^***^ | -.37^***^ | -.31^***^ | .33^***^ | .22^***^ | .30^***^ | .22^***^ | 1 |  |  |  |  |  |  |
|  | *N* | 4384 | 4373 | 4342 | 4276 | 4265 | 4359 | 2995 | 2971 | 1649 | 4216 | 1130 | 4334 | 3499 | 4382 | 4228 | 4349 | 4389 | 4385 | 4376 | 4391 | 4396 |  |  |  |  |  |  |
| Ethnicity | *r* | -0.03 | -.03^*^ | -.05^**^ | -0.03 | -.06^***^ | .05^**^ | 0.02 | 0.01 | 0.03 | .04^*^ | -0.01 | 0.02 | -0.02 | -.04^*^ | .07^***^ | .06^***^ | -.06^***^ | -.10^***^ | -.05^**^ | -0.01 | 0.01 | 1 |  |  |  |  |  |
|  | *N* | 4048 | 4040 | 4007 | 3946 | 3933 | 4025 | 2740 | 2711 | 1491 | 3892 | 1043 | 4001 | 3242 | 4049 | 3889 | 4012 | 4034 | 4032 | 4020 | 4040 | 3995 | 4062 |  |  |  |  |  |
| Gender | *r* | -.10^***^ | -.07^***^ | -.10^***^ | -.06^***^ | -.08^***^ | .06^***^ | -0.01 | 0.01 | -.09^***^ | 0.00 | -.09^**^ | .03^*^ | -.06^**^ | -0.01 | -0.01 | -.03^*^ | .04^**^ | 0.03 | .04^**^ | 0.01 | .08^***^ | 0.03 | 1 |  |  |  |  |
|  | *N* | 4458 | 4445 | 4415 | 4351 | 4337 | 4435 | 3051 | 3023 | 1671 | 4287 | 1143 | 4412 | 3558 | 4460 | 4261 | 4409 | 4432 | 4431 | 4417 | 4439 | 4380 | 4055 | 4474 |  |  |  |  |
| Education | *r* | 0.00 | -0.03 | 0.01 | .04^*^ | -.07^***^ | .04^**^ | -0.02 | -0.02 | 0.01 | 0.03 | 0.01 | .04^**^ | 0.02 | -.12^***^ | .12^***^ | .15^***^ | -.08^***^ | -.10^***^ | -.06^***^ | -.05^**^ | -.08^***^ | .08^***^ | .05^**^ | 1 |  |  |  |
|  | *N* | 4374 | 4364 | 4332 | 4270 | 4254 | 4350 | 2989 | 2964 | 1641 | 4206 | 1126 | 4329 | 3497 | 4375 | 4199 | 4330 | 4358 | 4356 | 4343 | 4364 | 4312 | 4007 | 4381 | 4389 |  |  |  |
| Age | *r* | .11^***^ | .17^***^ | .07^***^ | .06^***^ | .20^***^ | -.14^***^ | .05^*^ | 0.01 | -.05^*^ | -.04^**^ | -0.04 | .09^***^ | .10^***^ | .31^***^ | -.21^***^ | -.20^***^ | .15^***^ | .30^***^ | .20^***^ | .17^***^ | -.11^***^ | -.22^***^ | -.03^*^ | -.05^**^ | 1 |  |  |
|  | *N* | 4423 | 4412 | 4380 | 4316 | 4301 | 4401 | 3020 | 2994 | 1653 | 4255 | 1137 | 4377 | 3530 | 4425 | 4236 | 4379 | 4404 | 4404 | 4390 | 4412 | 4354 | 4041 | 4429 | 4369 | 4439 |  |  |
| Financial Situation | *r* | .13^***^ | .11^***^ | .10^***^ | .11^***^ | .05^**^ | -.06^**^ | 0.03 | -0.02 | 0.04 | -0.02 | 0.03 | 0.01 | .08^***^ | -.04^**^ | .27^***^ | .25^***^ | -.23^***^ | -.16^***^ | -.20^***^ | -.14^***^ | -.32^***^ | -.07^***^ | 0.01 | .21^***^ | .10^***^ | 1 |  |
|  | *N* | 3855 | 3844 | 3817 | 3762 | 3745 | 3826 | 2622 | 2595 | 1442 | 3704 | 1004 | 3809 | 3068 | 3849 | 3738 | 3819 | 3848 | 3843 | 3838 | 3849 | 3820 | 3567 | 3853 | 3835 | 3845 | 3861 |  |
| Born in Canada | *r* | 0.02 | 0.01 | 0.02 | -0.02 | .05^**^ | -.04^**^ | -.04^*^ | -0.00 | -0.03 | -0.00 | -0.03 | -.03^*^ | 0.00 | .05^**^ | -.08^***^ | -.08^***^ | .07^***^ | .06^***^ | .03^*^ | -0.02 | .10^***^ | -.47^***^ | -0.01 | -.16^***^ | .06^***^ | -0.02 | 1 |
|  | *N* | 4443 | 4431 | 4400 | 4336 | 4321 | 4420 | 3038 | 3011 | 1665 | 4274 | 1140 | 4397 | 3545 | 4445 | 4252 | 4397 | 4423 | 4423 | 4409 | 4431 | 4375 | 4058 | 4450 | 4384 | 4427 | 3859 | 4459 |

*Note. N* = 4485-647. Pearson and point biserial correlations are denoted with *r.*  ^***^*p* <.001, ^**^*p* <.01, ^*^*p* <.05. ^c^ denotes values that cannot be computed because at least one of the variables is constant. Gender coded as women and other gender groups = 1, men = 2. Several bivariate correlations could not be calculated, as these variables were dependent on each other, and thus not computable (i.e., the variables were constants). For example, patients were asked if they experienced any pain during their emergency department visit. If patients did experience pain, they were provided the question “during this emergency department visit, did the doctors and nurses try to help reduce your pain?”).
